# Chiroptical Characteristics of Nanosegregated Phases in Binary Mixture Consisting of Achiral Bent-Core Molecule and Bent-Core Base Main-Chain Polymer

**DOI:** 10.3390/polym14142823

**Published:** 2022-07-11

**Authors:** Ju-Yong Kim, Jae-Jin Lee, Suk-Won Choi

**Affiliations:** Department of Advanced Materials Engineering for Information & Electronics, Integrated Education Institute for Frontier Science & Technology (BK21 Four), Kyung Hee University, Yongin 17104, Korea; kjuyong0818@naver.com (J.-Y.K.); jjking1443@naver.com (J.-J.L.)

**Keywords:** bent-core molecules, polymers, chirality, liquid crystals, circular dichroism

## Abstract

In this paper, a binary mixture system consisting of an achiral bent-core molecule and a bent-core base main-chain polymer is described. The mixture exhibits an intriguing nanosegregated phase generated by the phase separation of the helical nanofilament B4 phase (originating from the bent-core molecule) and the dark conglomerate phase (originating from the bent-core base main-chain polymer). This nanosegregated phase was identified using polarized optical microscopy, differential scanning calorimetry, and X-ray diffraction analysis. In this nanosegregated phase, the enantiomeric domains grew to a few millimeters and a giant circular dichroism was observed. The structural chirality of the helical nanofilament B4 phase affected the conformation of the bent-core base main-chain polymer embedded within the helical nanofilament networks of bent-core molecules.

## 1. Introduction

Since the discovery of chiral resolution in achiral bent-core (BC) molecular systems [1], studies on such molecules have been actively conducted by chemists and physicists in the field of liquid crystal (LC) science [2,3,4,5,6,7,8,9,10,11]. The LC phases found in BC molecular systems differ from the conventional phases found in classic calamitic or discotic ones [12,13]. Among the LC phases in BC systems, the helical nanofilament (HNF) B4 phase has attracted significant interest owing to its unprecedented chiral superstructure, in which twisted nanofilaments are formed through the self-assembly of achiral molecules [14]. Subsequent studies considered the B4 phase to be semi-crystalline rather than LC [15]. However, the subject of chirality-related properties, such as strong optical activity and large effective nonlinear optical coefficients, has inspired several LC research groups [14].

Another intriguing symmetry-broken phase observed in achiral BC molecular systems is the dark conglomerate (DC) phase [5,14]. This phase is generally observed after direct cooling in the isotropic phase, which is optically isotropic and forms a macroscopic chiral domain. Although the optical appearance of the DC phase is similar to that of the B4 phase, the DC phase is fluid and can be considered to be a disordered lamellar phase resembling the lyotropic sponge (focal conic) phases, wherein the empty volume is filled by smectic layers with a saddle splay layer deformation [5,10,14]. The DC phase often emerges in BC molecules with a B2 or B7 phase and has been reported in BC polymeric systems [11].

Takanishi et al. reported that when a BC molecule exhibiting the B4 phase is mixed with a rod-like LC molecule, the B4 phase is stabilized preferentially over the other LC phases that appear in BC molecular systems, such as the B2 and B3 phases [16]. Furthermore, the chiral enantiomeric domains become significantly larger (up to a few millimeters larger in size) than those in the B4 phase of the pure BC molecule [16]. Subsequent studies have suggested that the phases in which the enantiomeric domains become unusually large are nanosegregated phases between the B4 phase (originating from BC molecules) and the calamitic phases (originating from rod-like molecules) [9,14]. Otani et al. examined the behavior of rod-like molecules in nanosegregated phases in which the rod-like molecules were embedded in the framework of the HNF B4 structure network, that is, a network of HNFs, formed by the BC molecules [17]. Based on circular dichroism (CD) observations, it was suggested that the rod-like molecules in the network of HNFs form a chiral superstructure that is affected by the HNF structure; thus, the chirality of the binary system can be boosted [17,18]. 

In this study, we performed chiroptical observations on a novel binary mixture consisting of a host BC molecule possessing the HNF B4 phase and a guest BC-based main-chain polymer (BCMP) possessing a DC phase. Binary systems composed of low-molecular BCs and BC-based polymers have rarely been investigated; therefore, details on the chiroptical properties of the intermediate phases of binary systems have also rarely been reported. Two different nanosegregated phases were identified in this binary system using polarized optical microscopy (POM), differential scanning calorimetry (DSC), and X-ray diffraction (XRD) analysis. In the nanosegregated phase observed in the high-temperature range, the BC molecule was in the B2 phase and the BCMP was in the DC phase. In the low-temperature nanosegregated phase, in which the BC molecule was in the HNF B4 phase and the BCMP was in the DC phase, the enantiomeric domains grew to a few millimeters in size, and a significantly strong CD intensity was observed.

## 2. Experimental Section

### 2.1. Materials

**Figure 1:**
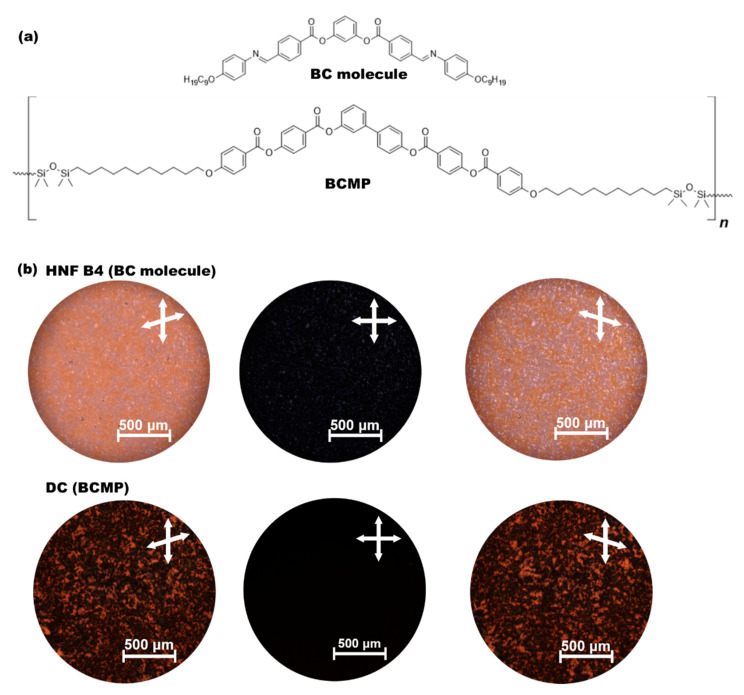
(**a**) Chemical structures of the BC molecule and BCMP used in this study. (**b**) Typical POM images of the HNF B4 phase (BC molecule) and DC phase (BCMP).

The chemical structures of the BC and BCMP used in this study are presented in Figure 1a. The BC molecule and BCMP were synthesized by our group. The BC and BCMP were synthesized using the methods reported by Akutakawa [19] and Gimeno [11], respectively. The phase sequences of the pure BC molecule and BCMP upon cooling were Iso-172 °C-B2-145 °C-B3-138 °C-B4 and Iso-168 °C-DC, respectively. The BC molecule and BCMP used contained the HNF B4 and DC phases in the low-temperature range, respectively. Figure 1b shows typical POM images of the HNF B4 phase (BC molecule) and DC phase (BCMP). Upon the uncrossing of the polarizer by moving it clockwise and counterclockwise by a few degrees, the interchanging of the colors of the two small, grainy domains in the HNF B4 phase corresponding to the BC molecule could be observed, indicating the existence of two enantiomeric domains with an almost identical degree of optical rotation but different signs [14]. Similarly, the two different-colored domains observed in the DC phase corresponding to the BCMP under uncrossing conditions suggest that the two enantiomeric domains had the same degree of optical rotation but different signs [14]. 

### 2.2. XRD Analysis

One-dimensional XRD measurements were performed employing a rotating-anode X-ray generator (Cu-Kα) and a diffractometer (Rigaku, Austin, TX, USA). The widths and peak positions of the diffraction angles were calibrated using silicon crystals. To monitor the changes in the local structure with respect to temperature, a hot stage calibrated with an error margin of ±1 °C was coupled to the diffractometer. The samples were scanned across the *q* range of 1–5 nm^−1^ at a scanning rate of 1 °C min^−1^.

### 2.3. CD Measurements

The mixture was injected into a sandwich cell (cell gap less than 2 μm) consisting of two quartz substrates. The inner surfaces of the cell were left untreated to prevent the phases from exhibiting a large birefringence. CD spectra were recorded using a CD spectrometer (J-815, Jasco, Hachioji, Japan). The CD signals were detected from an area of 1 mm in diameter.

## 3. Results

**Figure 2:**
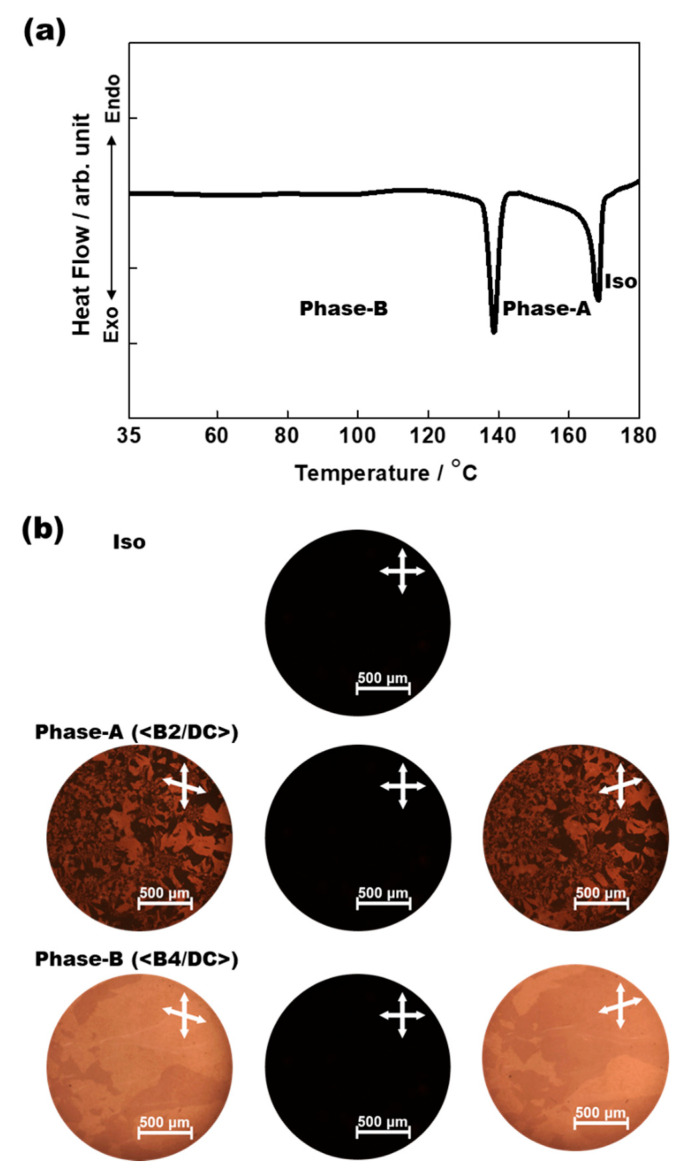
(**a**) Typical DSC profile obtained during the cooling of a binary mixture consisting of 70 wt% BC molecule and 30 wt% BCMP. (**b**) Typical POM images of Iso, Phase-A, and Phase-B.

Mixtures with different fractions of the BC molecule and BCMP were prepared. However, meaningful phases were not observed in the BCMP-rich systems because the DC phase was dominant. On the other hand, two distinct phases (Phase-A and Phase-B) were seen clearly in the BC molecule-rich systems using DSC and POM. Figure 2 shows the typical DSC profiles and POM images obtained during the cooling of the binary system, consisting of 70 wt% BC and 30 wt% BCMP. Two distinct peaks were detected in the DSC profile, as shown in Figure 2a, and the typical POM images for the corresponding temperature range are given in Figure 2b. POM images were observed with Eclipse E600W (Nikon, Tokyo, Japan) equipped with a LTSE420 temperature control unit (Linkam, Redhill, UK). When the temperature was lowered from that corresponding to the Iso phase, which exhibited a perfectly dark state under crossed polarizers, a different phase (Phase-A) distinct from the Iso phase appeared; two different dark-colored domains were observed when the upper polarizer was rotated slightly clockwise (or anticlockwise). The two colors switched under opposite uncrossing conditions. Upon further cooling, another phase (Phase-B) with a higher brightness was observed; the sizes of the two-colored domains were larger in this domain. Interestingly, the domains observed in Phase-B were significantly larger the domains observed in Phase-A, and they became larger, up to a few millimeters in size. These unusual domain sizes in Phase-B are comparable to those seen in the nanosegregated phases of mixed systems consisting of BC and rod-like molecules.

**Figure 3:**
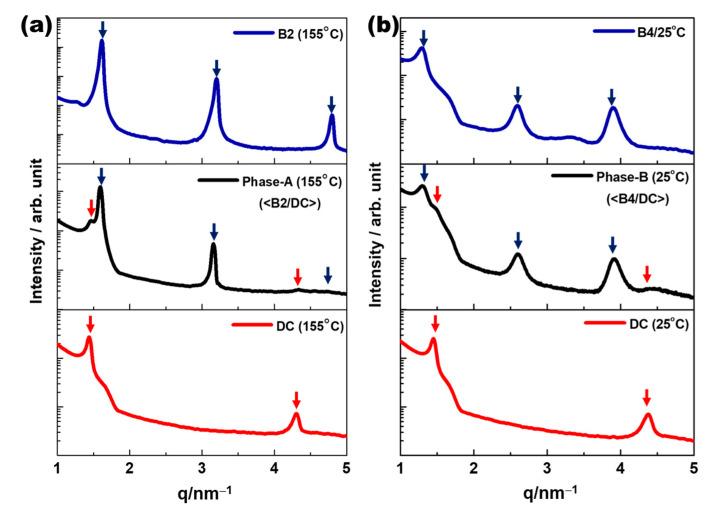
(**a**) A typical small-angle-region XRD profile obtained over a temperature range corresponding to Phase-A and XRD profiles of pure B2 (originating from BC molecules) and pure DC (originating from BCMP). (**b**) A typical small-angle-region XRD profile obtained over a temperature range corresponding to Phase-B and XRD profiles of pure HNF B4 (originating from BC) and pure DC (originating from BCMP).

**Figure 4:**
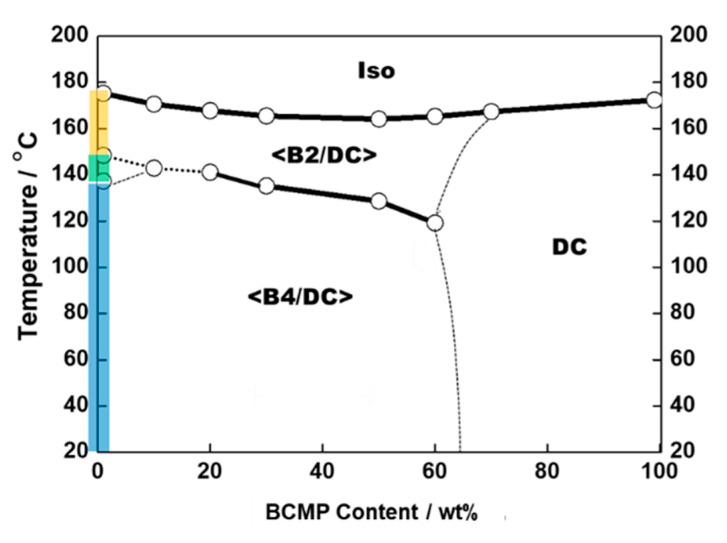
Phase diagram of various mixtures containing different fractions of BC molecule and BCMP.

XRD measurements were performed to elucidate the local structures of Phase-A and Phase-B. Figure 3a shows a typical XRD profile in the temperature range of Phase-A for a small-angle region, and the XRD profiles of pure B2 (originating from the BC molecule) and pure DC (originating from the BCMP). In the pure B2 phase, three distinct diffraction peaks were detected (at 1.3, 2.6, and 3.9 nm^−1^), while in the pure DC phase, two distinct peaks were observed (at 1.45 and 4.37 nm^−1^). As shown in Figure 3a, the XRD profile for Phase-A could be considered to be a simple superposition of those for the pure B2 and DC phases. Thus, Phase-A segregated the <B2/DC> nanophase, whereas the BC and BCMP were in the B2 and DC phases, respectively. Figure 3b exhibits a typical XRD profile in the temperature range of Phase-B for a small-angle region along with the XRD profiles of pure HNF B4 (originating from the BC) and pure DC (originating from the BCMP). In the pure B4 phase, three distinct diffraction peaks were observed (at 1.6, 3.2, and 4.8 nm^−1^). The XRD pattern of Phase-B also appeared to be a superposition of those of HNF B4 (BC) and DC (BCMP). Thus, Phase-B segregated the <B4/DC> nanophase, in which the BC and BCMP were in the HNF B4 and DC phases, respectively. Based on the results of the above-mentioned DSC, POM, and XRD analyses, upon cooling, the binary system consisting of 70 wt% BC molecule and 30 wt% BCMP was assigned the structure Iso-168 °C-<B2/DC>-140 °C-<B4/DC>. A phase diagram of the various mixtures with different fractions of the BC molecule and BCMP is shown in Figure 4. 

**Figure 5:**
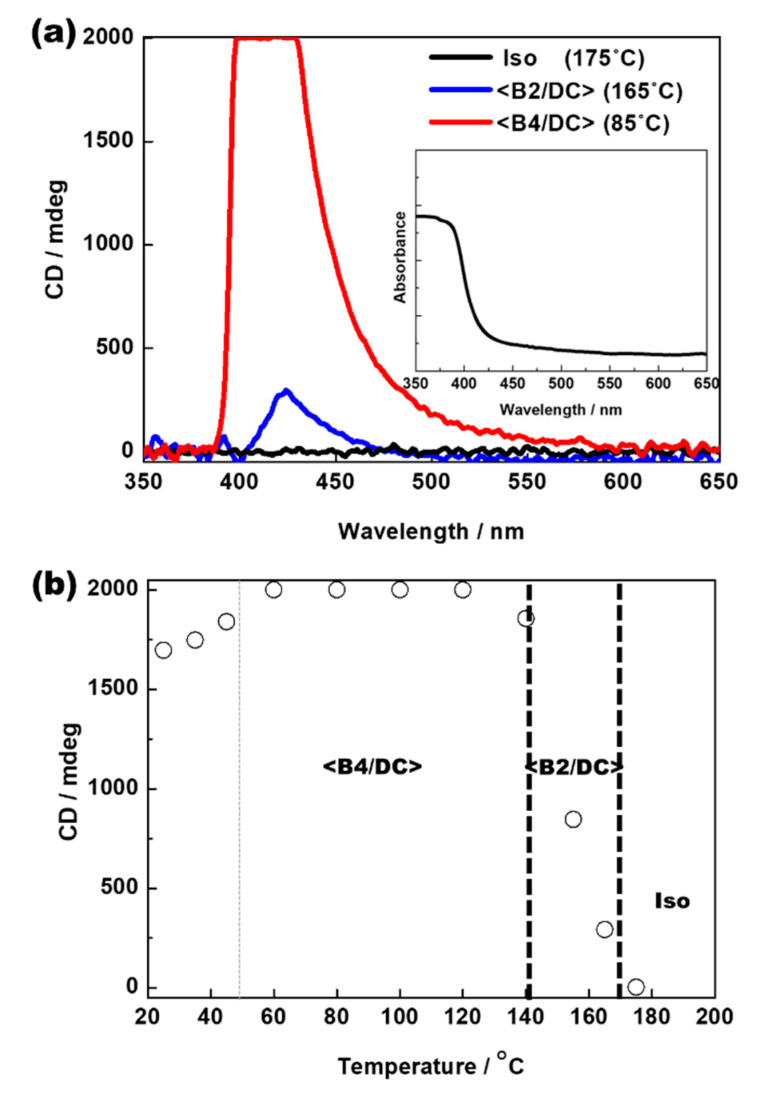
(**a**) Typical induced CD spectra from positive enantiomeric domains in Iso, <B2/DC>, and <B4/DC> in a binary mixture consisting of 70 wt% BC molecule and 30 wt% BCMP (inset: absorbance spectrum of binary mixture) and (**b**) maximum CD peak intensity of positive domain at approximately 415 nm as a function of temperature.

Figure 5a shows the typical CD spectra induced from the positive enantiomeric domains in Iso, <B2/DC>, and <B4/DC> for the binary system consisting of 70 wt% BC molecule and 30 wt% BCMP. The absorbance spectrum of the binary system is shown in the inset of Figure 5a. Figure 5b shows the maximum CD peak intensity of the positive domain at approximately 415 nm as a function of temperature. Intriguingly, a small change in the peak intensity was observed; this occurred within the phase-transition temperatures of the binary mixture. Significant CD signals were detected in <B2/DC>, and these increased gradually when the temperature decreased. Finally, the CD signals detected in <B4/DC> were significantly larger than those seen in <B2/DC>. 

## 4. Discussion

**Figure 6:**
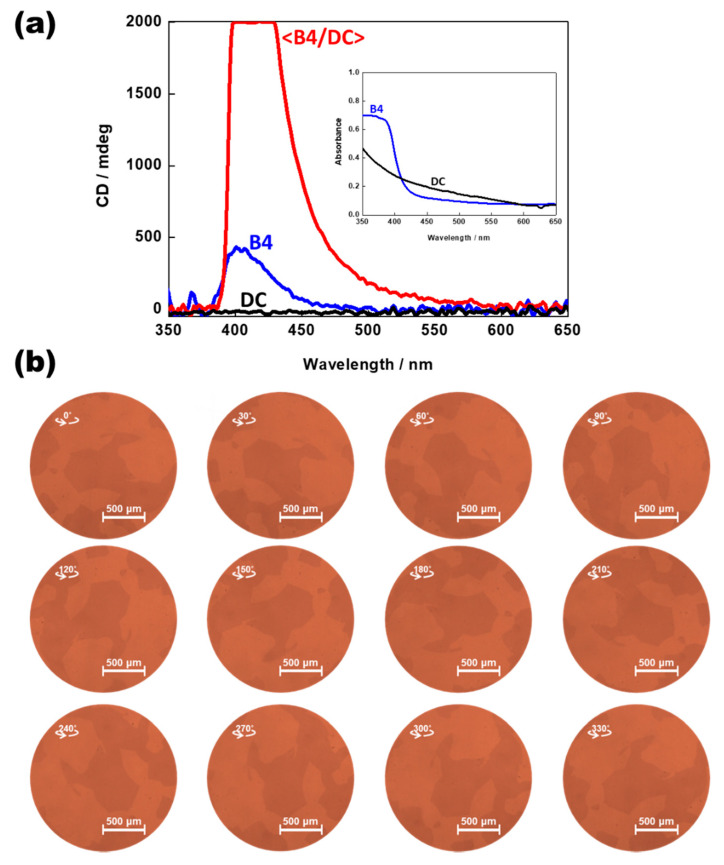
(**a**) Typical induced CD spectra of pure HNF B4 (BC molecule), pure DC (BCMP), and <B4/DC> (binary mixture) (inset: absorbance spectra of pure HNF B4 (BC molecule) and pure DC (BCMP)). (**b**) POM images of <B4/DC> taken by rotating the cell about cell surface normal under uncrossing conditions.

We focused on the nanosegregated <B4/DC> phase with unusually large enantiomeric domains (a few millimeters in size) and giant CD signals. Figure 6a shows a comparison of the typical induced CD spectra of pure HNF B4 (BC molecule), pure DC (BCMP), and <B4/DC> (binary mixture). The absorbance spectra of the BC molecule and BCMP are presented in the inset of Figure 6a. A large optical rotation was predicted for the DC phase [5]; however, the observed CD signals were negligible in this case. This was because the observed spot size (1 mm in diameter) was larger than that of the enantiomeric domains (a few hundred micrometers) of the DC phase. Although a single enantiomeric domain possesses a large optical rotation, the two small enantiomeric domains with almost the same degree of optical rotation but different signs, and observed within the same spot size, canceled each other out. However, in <B4/DC>, the sizes of the enantiomeric domains increased to a few millimeters (see Figure 2b), and a significantly strong CD intensity signal was observed, as shown in Figure 6a. The small (or negligible) CD signals of HNF B4 (or DC) were amplified by the nanosized separation between HNF B4 and DC. The effect of linear birefringence in <B4/DC> should be considered because the observed CD is the sum of the actual CD and the sample birefringence [18]. To confirm that the linear birefringence did not contribute to the CD signal, POM images were taken while rotating the cell about the cell surface normal under uncrossing conditions. As shown in Figure 6b, the two colors attributable to the two enantiomeric domains with almost the same degree of optical rotation but different signs were not inverted by the rotation of the cell. Thus, the significantly strong CD signals of <B4/DC> are attributable to actual CD and not to linear birefringence. 

**Figure 7:**
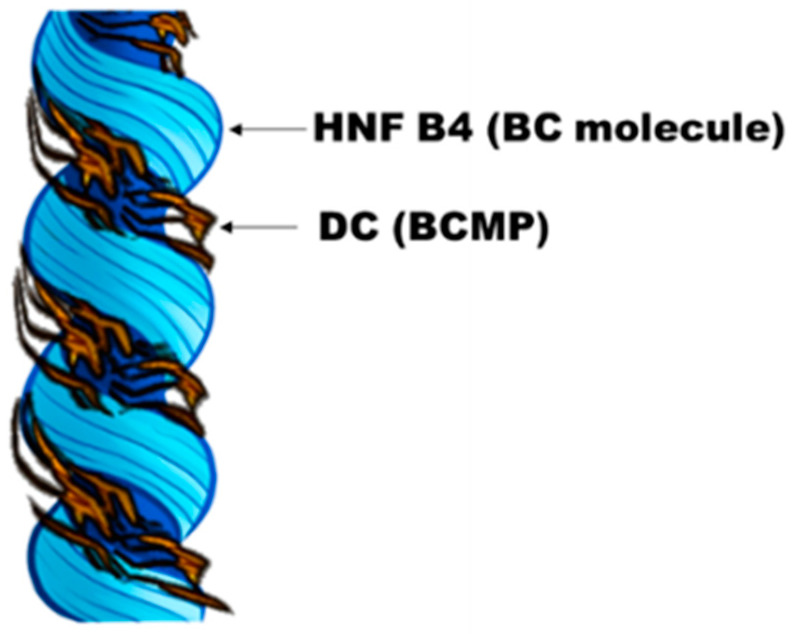
Proposed model of the chiral superstructure in the <B4/DC>, assuming that the polymer chains of BCMP align in parallel to the groove of the HNFs.

These unusual phenomena observed in <B4/DC> are similar to those reported in the nanoseparation phases in a mixture consisting of BC and calamitic molecules [17,18]. The structural chirality of HNF B4 affects the conformation of the BCMP embedded within the HNF B4 networks of the BC molecules, causing the BCMP to exhibit a chiral conformation, as shown in Figure 7. In addition, the structural chirality in HNF B4 was amplified by the DC medium of the BCMP. Thus, the flexibility of the polymeric chains consisting of DC affected the chiral conformation of the BCMP. As shown in Figure 5b, at a temperature of 50 °C, which is the T_g_ of the BCMP, the CD intensity in <B4/DC> decreased. This is because the effect of the structural chirality of HNF B4 on the conformation of the BCMP was weakened by the lowered flexibility of the polymer chains in the DC phase. 

## 5. Conclusions

Chiroptic observations were performed on a novel binary mixture consisting of a BC molecule possessing an HNF B4 phase and a main-chain polymeric material possessing a DC phase. Two distinct nanosegregated phases were observed in the binary system. In the <B2/DC> phase, observed in the high-temperature range, the BC molecule was in the B2 phase and the BCMP was in the DC phase. In contrast, another <B4/DC> phase was observed in the low-temperature range, in which the BC molecule was in the HNF B4 phase and the BCMP was in the DC phase. In <B4/DC>, the enantiomeric domains grew to a few millimeters, and a significantly strong CD intensity was observed. We confirmed that these CD signals were not attributed to linear birefringence but to actual CD. It is likely that the HNF B4 networks of the BC molecules play an important role in the unusual phenomena observed in this system. The structural chirality of HNF B4 affects the conformation of the BCMP embedded within the HNF B4 networks of the BC molecules, conferring the BCMP a chiral conformation. The mesophase systems exhibiting spontaneous phase separation and hierarchical molecular organization presented in this study should lead to the emergence of novel chiroptical materials. 

## Data Availability

The data presented in this study are available on request from the corresponding author.
